# Serum Exosomal MicroRNA-21, MicroRNA-126, and PTEN Are Novel Biomarkers for Diagnosis of Acute Coronary Syndrome

**DOI:** 10.3389/fphys.2020.00654

**Published:** 2020-06-11

**Authors:** Hao Ling, Ziyuan Guo, Yongfeng Shi, Lei Zhang, Chunli Song

**Affiliations:** ^1^Department of Cardiology, The Second Hospital of Jilin University, Changchun, China; ^2^Department of Neurology, The Second Hospital of Jilin University, Changchun, China

**Keywords:** exosomal miRNA-126, exosomal miRNA-21, PTEN, coronary heart disease, acute coronary syndrome, biomarker severity, coronary artery stenosis

## Abstract

Acute coronary syndrome (ACS) is a serious threat to public health. Based on clinical manifestations, ACS can be classified into unstable angina (UA) pectoris and acute myocardial infarction (AMI). The purpose of this study was to explore the possibility of using serum exosomal microRNA (miR)-126, miR-21, and phosphatase and tensin homolog (PTEN) expression levels as biomarkers of UA and AMI and to investigate whether these levels were positively correlated with the severity of coronary stenosis based on the Gensini score. Exosomes were isolated by ultracentrifugation from the serum of 34 patients with AMI, 31 patients with UA, and 22 healthy controls. The isolated exosomes were characterized by electron microscopy and particle size analysis; exosomal identity was further confirmed by western blotting using exosome-specific antibodies. Real-time quantitative polymerase chain reaction indicated that the serum exosomal levels of miR-126 and miR-21 were significantly higher in the patients with UA and AMI than in the healthy controls. Enzyme-linked immunosorbent assay showed that the serum exosomal PTEN levels were significantly higher in the UA and AMI groups than in the control group. Receiving operating characteristic curve analysis demonstrated the diagnostic efficiency of serum exosomal miR-126, miR-21, and PTEN levels for predicting AMI and UA. In addition, the circulating exosomal miR-126 level was positively correlated with the severity of coronary artery stenosis in patients with UA and AMI based on the Gensini score.

## Introduction

Coronary heart disease is the main cause of death in adults in the United States, accounting for one-third of all deaths among adults over 35 years old (Sanchis-Gomar et al., 2016). Acute coronary syndrome (ACS) is the most severe type of coronary heart disease and is a life-threatening condition, with 625,000 cases diagnosed globally in 2010 (Mozaffarian et al., 2015) which increased up to 633,000 by 2014 (Benjamin et al., 2019). ACS can be classified as unstable angina (UA) pectoris and acute myocardial infarction (AMI). Traditionally, the diagnosis of AMI is based on clinically abnormal levels of certain cardiac biomarkers, including troponin, which is also a cardiac biomarker acute myocardial ischemia. However, it takes 3–4 h for the troponin level to increase after the occurrence of myocardial infarction. Therefore, the critical window for the clinical treatment of AMI may be missed. In addition, the increase of cardiac troponin indicates that cardiac myocytes are damaged (Roffi et al., 2016). At present, it has been found that patients with heart failure, hypertension and cardiac perioperative period also have the phenomenon of troponin increase (Kociol et al., 2010; Roffi et al., 2016; Lionetti and Barile, 2020) so troponin is not specific enough in term of the diagnosis of AMI. Meanwhile, there is still no reliable biomarker for the diagnosis of UA.

MicroRNAs (miRNAs, miRs) are a group of small non-coding single-stranded RNAs of ∼22 nucleotides in length, which are highly evolutionarily conserved and exist in almost all eukaryotes. MiRNAs play important roles in almost all cellular processes (Bartel, 2004) with accumulating evidence showing their important roles in coronary heart disease (Small and Olson, 2011; Welten et al., 2016; Widmer et al., 2016). Recently, many studies have focused on miRNAs in an attempt to identify UA- and AMI-related biomarkers (Gholamin et al., 2016). For example, circulating miR-17-5p, miR-126-5p, and miR-145-3p have been identified as new biomarkers for AMI (Xue et al., 2019). Moreover, a variety of miRNAs, including other nucleic acid molecules such as other non-coding RNAs and mRNAs have been found in exosomes (Zhang et al., 2015) which are nanoscale vesicles (40–100 nm in diameter) that can be released into the extracellular space from a variety of cell types and are widely distributed in various bodily fluids. When exosomes circulate, exosomal RNAs can be taken up by adjacent or distant cells to regulate the activity of recipient cells. Exosomes are highly stable in biological fluids because the packaging in a lipid bilayer can prevent them from being degraded by humoral enzymes, resulting in a relatively long and stable expression duration (Chen et al., 2017). Therefore, research on exosomes has increasingly potential for the diagnosis and treatment of diseases. For example, increased levels of exosomal miR-223 were associated with the occurrence of acute ischemic stroke, as well as with the severity and short-term prognosis of stroke (Chen et al., 2017). Moreover, exosomal miRNAs are currently used as biomarkers and therapeutic targets for cancer (Thind and Wilson, 2016).

The phosphatase and tensin homolog (*PTEN*) gene on chromosome 10 is a tumor-suppressor gene that plays an important role in inhibiting the proliferation and migration of many types of cancer cells (Marsh et al., 1997). A recent study showed that miR-21 can promote the proliferation of smooth muscle cells and the formation of atherosclerotic plaques by inhibiting the PTEN-induced activation of AKT (Zhang et al., 2019), which had protective effects against endothelial injury after AMI through the PTEN/vascular endothelial growth factor pathway (Yang et al., 2016). Similarly, miR-126 was reported to inhibit the apoptosis of vascular endothelial cells by targeting PI3K/AKT signal transduction (Chen et al., 2016). Exosomal miR-21 from mesenchymal stem cells was also shown to mediate cardioprotective effects after AMI (Luther et al., 2018). However, the potential of the exosomal levels of miR-21 and miR-126, as well as those of their downstream signaling protein PTEN, as diagnostic markers for AMI and UA, and the relationship between these markers and the severity of AMI and UA have not yet been investigated. The use of exosomal miRNA levels and respective disease scores (such as the Gensini score; Gensini, 1983) would offer an improved method for non-invasively estimating the degree of coronary artery stenosis to facilitate the rapid diagnosis and treatment of patients with ACS.

Therefore, in this study, we investigated whether the exosomal levels of miR-21, miR-126, and PTEN extracted from patient serum samples can be used as diagnostic markers for AMI and UA and whether these indices are related to the severity of coronary artery stenosis.

## Materials and Methods

### Ethics Statement

This study was carried out at The Second Hospital of Jilin University in Changchun, China. Blood samples were collected between June and October 2019 from patients at the Department of Cardiovascular Medicine and from a healthy population that underwent routine physical examinations of the Second Hospital of Jilin University. The study was conducted in accordance with the principles of the Declaration of Helsinki. The study design was approved by the ethical committee of The Second Hospital of Jilin University (2019062), and the study was registered with the Chinese Clinical Trial Registry (ChiCTR1900026192).

### Participants

All subjects voluntarily participated in this study and provided written informed consent. Age, sex, course of disease, and other general information was recorded for all subjects. Baseline laboratory characteristics of all subjects were detected by enzyme-linked immunosorbent assay (ELISA) at the clinical Laboratory of the Second Hospital of Jilin University.

A total of 34 patients with AMI, 31 patients with UA, and 22 healthy controls were selected for inclusion in the study. The AMI group was established based on the diagnosis of AMI in accordance with the fourth edition of the global unified definition of myocardial infarction, released at the annual meeting of the European Society of Cardiology (Benjamin et al., 2019). Abnormal levels of cardiac biomarkers confirmed acute myocardial damage and served as clinical evidence of AMI. The inclusion criteria for subjects in the UA pectoris group were as follows: (1) increase in the severity and frequency of the original stable angina pectoris, and prolonged duration of angina pectoris; (2) angina pectoris occurred during periods of rest; and (3) angina pectoris was induced by mild physical activity in the last month. A diagnosis of UA pectoris was also established if the subject had a history of stable angina pectoris, myocardial infarction, and abnormal coronary angiography, and showed positive exercise test results even without a corresponding ST-T change in the electrocardiogram, but with typical UA pectoris symptoms. Completely healthy adults, with a similar age to that of the subjects in the UA and AMI groups were selected to be part of the healthy control group.

The exclusion criteria for participation were as follows: (1) presence of infection, inflammation, or autoimmune diseases, progressive hepatic and renal insufficiency, and tumor history; (2) under 40 and over 70 years of age; (3) blood glucose >7.0 mmol/L; (4) systolic blood pressure <90 or >140 mmHg; (5) diastolic blood pressure >90 mmHg; (6) patients with UA and AMI who did not undergo coronary angiography on the day after the blood samples were drawn.

### Criteria for Coronary Artery Stenosis and Gensini Score

The evaluation of coronary angiography was carried out by two experienced doctors, and the position of coronary angiography was based on the principle of fully exposed lesions. The stenosis of each branch of the coronary artery was recorded and scored using the Gensini scale (Gensini, 1983).

### Sample Collection and Storage

In this study, 5 mL fasting blood samples were collected from the subjects in the UA and healthy control groups before breakfast, and within 2 h after AMI from the subjects in the AMI group. After centrifugation (Z 366; Hermle Labortechnik GmbH, Germany) at 4°C and 3,000 *g* for 10 min, the serum was collected and stored in a 1.5-mL RNAse-free EP tube at −80°C. Exosomes were isolated from the serum by ultracentrifugation (XPN-80; Beckman Coulter, United States) at 110,000 *g* for 80 min at 4°C. The supernatant was discarded, and the precipitate was thoroughly resuspended in 9 mL of phosphate-buffered saline (PBS). The ultracentrifugation was repeated under the same condition, and after discarding the supernatant, 200 μL of PBS was added to the precipitate to prepare an exosome suspension, which was transferred to a 1.5 mL EP tube for preservation.

### Electron Microscopy and Particle Size Analysis

For electron microscopic analysis, 5 μL of the exosomal suspension using PBS was diluted to 10 μL. The sample was placed onto a copper grid, and after 1 min, excess liquid was blotted with a filter paper. Then, 10 μL of a 2% phosphotungstate solution was added to the copper grid, left to absorb for 1 min, and filter paper was used to blot dry the excess liquid. The samples were dried at room temperature for several minutes and then observed under an electron microscope (HT-7700; Hitachi, Japan) at 80 kV.

For particle size analysis, 5 μL of the exosomal suspension using PBS was diluted to 30 μL, and the particle size and concentration of exosomes were analyzed using a Flow NanoAnalyzer (NanoFCM, Xiamen, China) according to the manufacturer’s instructions.

### Western Blotting

Exosomal samples (20 mg) were lysed on ice in 150–250 μL of RIPA buffer (Solarbio, Beijing, China). The samples were then centrifuged (Z 366) at 4°C and 3,000 *g* for 15 min, and protein concentrations of the supernatants were quantified using a bicinchoninic acid (BCA) assay kit (Thermo Fisher Scientific, Waltham, MA, United States). Western blotting was then performed with a Solarbio western blotting kit according to the manufacturer instructions. In brief, the protein samples were electrophoresed on a 10–15% sodium dodecyl sulfate-polyacrylamide gel electrophoresis gel (with 15 μg of protein per well), and the separated proteins were transferred onto polyvinylidene fluoride membranes, which were then blocked with 5% milk in Tris-buffered saline with Tween 20 blocking buffer. The membranes were incubated with antibodies against CD9 (Abcam, ab19715, 1:1000 dilution), CD63 (Abcam, ab59479, 1:1000 dilution), and TSG101 (Abcam, ab125011, 1:1000 dilution) overnight at 4°C. Following incubation with the specific horseradish peroxidase-conjugated goat anti-rabbit IgG secondary antibody (Thermo Fisher Scientific, MA1108HRP, 1:1000 dilution), the chemiluminescence signal was detected using ECL western blotting detection reagents (Millipore, WBKLS0100, New York, NY, United States).

### Extraction of Total RNA

The exosomes from the serum (200 μL) were mixed with 250 fm cel-miR-39-3p (Tiangen Biotech, Beijing, China) and 1 mL of TRIzol reagent (Invitrogen, Carlsbad, CA, United States). This mixture was homogenized for 20 s and then immediately placed on ice for 5 min, followed by centrifugation at 12,000 *g* for 10 min. The supernatant was collected into a new 1.5 mL centrifuge tube and thoroughly mixed with 200 μL of chloroform. After incubation for 2 min at 4°C, the sample was centrifuged at 12,000 *g* for 10 min at room temperature. The supernatant was transferred to a new 1.5-mL centrifuge tube, followed by mixing with 600 μL of isopropanol. This mixture was incubated for 15 min at 4°C and then centrifuged at 12,000 *g* for 15 min at room temperature. The supernatant was discarded, and then 1 mL of 75% ethanol was added to the samples, followed by centrifugation at 12,000 *g* for 5 min at 4°C, and the supernatants were discarded. Then, 40 μL aliquots of DEPC water were added to dissolve the RNA, and the samples were stored at −80°C for further analysis.

### Polyadenylation and Reverse Transcription of miRNA

The complementary DNA (cDNA) was synthesized using a reverse transcription (RT) kit (Fermentas, Waltham, MA, United States) according to the manufacturer’s protocol. The total reaction volume was 25 μL, including 12 μL of RNA/primer mix, 5 μL of 5 × RT reaction buffer, 1 μL of 25 mM dNTPs, 1 μL of an RNase inhibitor (25 U/μL), 1 μL of RT primers, and 3 μL of ddH_2_O (DNase-free). The reaction was performed as follows: 37°C for 60 min, 85°C for 5 min, and 4°C for 5 min. The following RT primers were used: *Homo sapiens* miR-21 (5′-CTCAACTGGTGTCGTGGAGTCGGCAATTCAGTTGAG ACAGCC-3′) and *H. sapiens* miR-126 (5′-CTCAACTGGTGTC GTGGAGTCGGCAATTCAGTTGAGCGCGTA-3′).

### Real-Time PCR Amplification

Real-time PCR amplification was performed using a SYBR Green PCR kit (Thermo Fisher Scientific) according to the manufacturer’s protocol. The cycling conditions were as follows: initial denaturation at 95°C for 10 min, followed by 40 cycles at 95°C for 15 s and 60°C for 45 s, one cycle at 95°C for 15 s and 60°C for 1 min, and a final cycle at 95°C for 15 s and 60°C for 15 s. The data were analyzed using ABI Prism 7300 SDS software (Applied Biosystems, Foster City, CA, United States). Each experiment was repeated three times, and relative expression levels of miRNA were analyzed using the 2^–ΔΔCt^ method (Xue et al., 2019). The sequences of the primers used were as follows: *H. sapiens* miR-21F (5′-ACACTCCAGCTGGGCAACACCAGTCGATG-3′) and miR-21R (5′-TGGTGTCGTGGAGTCG-3′); *H. sapiens* miR-126F (5′-ACACTCCAGCTGGGCATTATTACTTTTGG-3′) and miR-126R (5′-TGGTGTCGTGGAGTCG-3′); and *H. sapiens* endogenous U6 RNA-F (5′-CTCGCTTCGGCAGCACA-3′) and U6 RNA-R (5′-AACGCTTCACGAATTTGCGT-3′) as a normalization reference.

### ELISA

Protein concentrations were quantified in the exosomal supernatants using a BCA assay kit (Thermo Fisher Scientific). Five micrograms of total protein from exosomal supernatants was diluted in DEPC water to 50 μL, which was used as the sample for colorimetric ELISA with the Human PTEN ELISA kit (Biovision, XY-K4193-100, SFO, United States) according to the manufacturer’s instructions. The absorbance of the samples was measured at 450 nm using a Multiskan MS microplate reader (Labsystems Diagnostics Oy, Finland). The linear regression equation of the standard curve was calculated after plotting the concentrations vs. absorbance values of samples. The absorbance value of a sample was used to calculate its concentration from the standard curve, and then the concentration was multiplied by the dilution factor to obtain the actual concentration of the sample, which reflected the expression of serum exosomal PTEN.

### Statistical Analysis

All patients’ clinical characteristics are presented as the means ± standard deviations (SDs). Multiple comparisons of categorical variables were conducted using a chi-squared test, and ordinal variables were compared using analysis of variance (ANOVA). For data with a non-parametric distribution, the Kruskal–Wallis test was used, followed by the Mann–Whitney *U*-test. Pearson’s correlation coefficient was used to validate the relationships between continuous variables. Statistical analyses were conducted using GraphPad Prism 8.0 (GraphPad Software, San Diego, CA, United States) and SPSS 24 (IBM, New York, NY, United States). The threshold for statistical significance was set at *P* < 0.05.

## Results

### Baseline Characteristics of the Study Population

Table 1 summarizes the baseline characteristics of the 22 healthy subjects in the control group, the 34 patients with AMI, and the 31 patients with UA. Except for NT-proBNP, no other variables showed significant differences among the three groups. Significant differences in NT-proBNP levels were observed both between the UA and control groups and between the AMI and control groups.

**TABLE 1:** Baseline data of the study subjects.

Variable	UA group	AMI group	Control group	*P*-value
Male	19	24	11	0.299^a^
Female	12	10	11	
Age (years)	60.138.26	57.768.51	58.55.27	0.462^b^
HR (beats/min)	74.1912	77.3813.28	72.867.91	0.323^b^
SBP (mmHg)	132.2311.16	127.4715.02	128.456.23	0.257^b^
DBP (mmHg)	80.6810.05	82.7611.9	76.238.82	0.081^b^
Cr (μmol/L)	81.8128.2	82.8223.4	80.3613.29	0.928^b^
NT-proBNP	119.4122.48	148.22156.6	46.9521.01	0.013^b^
Glu (mmol/L)	5.721.15	5.960.78	5.880.6	0.547^b^
TG (mmol/L)	1.70.83	1.581.05	1.520.61	0.738^b^
TC (mmol/L)	4.090.96	3.91.08	3.520.58	0.096^b^
LDL (mmol/L)	2.590.82	2.640.73	2.730.67	0.796^b^
HDL (mmol/L)	1.010.25	1.070.22	1.170.32	0.081^b^
cTnI (ng/mL)	0.140.57	1.411.14	0.060.31	0.001^b^

*HR, heart rate; SBP, systolic blood pressure; DBP, diastolic blood pressure; Cr, creatinine; NT-proBNP, N-terminal pro-B natriuretic peptide; Glu, blood glucose; TG, triglyceride; TC, total cholesterol; LDL, low-density lipoprotein; HDL, high-density lipoprotein; cTnI, cardiac troponin I. Data are shown as the means ± SDs. ^*a*^Chi-squared test. ^*b*^ANOVA.*

### Characterization of Exosomes

**FIGURE 1:**
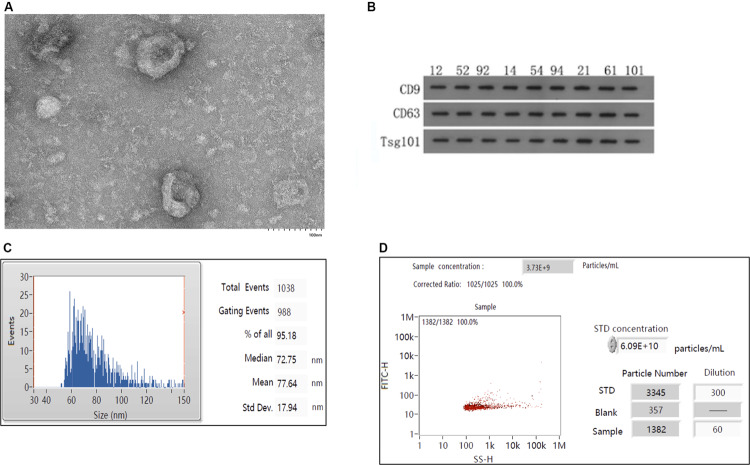
Isolation and characterization of exosomes. **(A)** Electron mirror diagram of exosome extraction. **(B)** Results of western blotting of serum exosomal samples (*n* = 9). Numbers marked on the strip indicate randomly selected samples. **(C)** Analysis of particle sizes of exosomes. **(D)** Analysis of exosomal concentrations.

As shown in Figure 1A, the exosomes extracted from the serum by ultracentrifugation were fully purified and had a tea tray-like structure. As shown in Figure 1B, the identity of the exosomes was confirmed by western blotting using antibodies against the exosome-specific marker proteins CD9, CD63, and TSG101. The average diameter of the isolated exosomes was 77.64 nm, with a median diameter of 72.75 nm and standard error of 17.94 nm (Figure 1C). The proportion of particles in the 30–150 nm size range was 95.18%, which corresponds to the typical diameter of exosomes. As shown in Figure 1D, the concentration of exosomes obtained was 3.73 × 10^9^/mL.

### Serum Exosomal Expression of miR-21, miR-126, and PTEN in Different Groups

**FIGURE 2:**
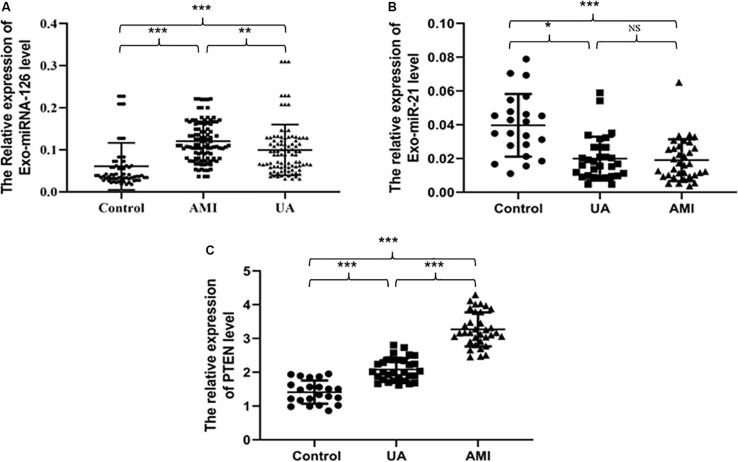
Relative serum exosomal expression levels of miR-126 **(A)**, miR-21 **(B)**, and PTEN **(C)** in samples from different groups. *P < 0.05, **P < 0.01, ***P < 0.001.

Figure 2 and Tables 2–4 show that the expression levels of miR-126 and PTEN in serum exosomes from the AMI and UA groups were significantly higher than those in the healthy control group, and the expression levels in the AMI group were significantly higher than those in the UA group. By contrast, the expression level of miR-21 in serum exosomes from the AMI and UA groups was significantly lower than that in the healthy control group and did not differ significantly between the AMI and UA groups.

**TABLE 2:** Comparison of relative serum exosomal miR-126 expression levels among groups.

Group (I)	Group (J)	Mean deviation (I–J)	SE	*P-*value	95% Confidence interval	
					Lower	Upper
Control	AMI	–0.059668317	0.008782367	0.001	–0.07696257	–0.04237407
	UA	–0.038830662	0.008947755	0.001	–0.05645059	–0.02121073
AMI	Control	0.059668317	0.008782367	0.001	0.04237407	0.07696257
	UA	0.020837655	0.007970851	0.009	0.00514144	0.03653387
UA	Control	0.038830662	0.008947755	0.001	0.02121073	0.05645059
	AMI	–0.020837655	0.007970851	0.009	–0.03653387	–0.00514144

*AMI, acute myocardial infarction; UA, unstable angina pectoris.*

**TABLE 3:** Comparison of relative serum exosomal miR-21 expression among groups.

Group (I)	Group (J)	Mean deviation (I–J)	SE	*P-*value	95% Confidence interval	
					Lower	Upper
Control	AMI	–0.0596683167	0.0150839401	0.001	–0.089664386	–0.029672248
	UA	–0.0388306618	0.0153679993	0.013	–0.069391614	–0.008269710
AMI	Control	0.0596683167	0.0150839401	0.001	0.029672248	0.089664386
	UA	0.0208376549	0.0136901421	0.132	–0.006386694	0.048062004
UA	Control	0.0388306618	0.0153679993	0.013	0.008269710	0.069391614
	AMI	–0.020837655	0.007970851	0.009	–0.03653387	–0.00514144

*AMI, acute myocardial infarction; UA, unstable angina pectoris.*

**TABLE 4:** Comparison of relative serum exosomal PTEN expression among groups.

Group (I)	Group (J)	Mean deviation (I–J)	SE	*P-*value	95% Confidence interval	
					Lower	Upper
Control	AMI	–1.857804128	0.1126966195	0.001	–2.081914400	–1.633695226
	UA	–0.6708636364	0.1148189106	0.001	–0.899193632	–0.442533641
AMI	Control	1.8578048128	0.1126966195	0.001	–2.081914400	–1.633695226
	UA	1.1869411765	0.1022831389	0.001	0.983539938	1.390342415
UA	Control	0.6708636364	0.1148189106	0.001	–0.899193632	–0.442533641
	AMI	–1.1869411765	0.1022831389	0.001	0.983539938	1.390342415

*AMI, acute myocardial infarction; UA, unstable angina pectoris.*

### Correlation and Multivariate Analyses

We used Spearman’s correlation analysis to identify potential factors associated with the expression levels of serum exosomal miR-21, miR-126, and PTEN and found no significant correlations that could affect the observed associations (Table 5).

**TABLE 5:** Correlations between serum exosomal miR-21, miR-126, and PTEN levels and classical cardiovascular risk factors in patients with acute myocardial infarction (AMI) and unstable angina pectoris (UA) and control subjects.

Variable		Exosomal miR-126			Exosomal miR-21			Exosomal PTEN		
		Control	UA	AMI	Control	UA	AMI	Control	UA	AMI
Age	*r*	–0.181	0.082	–0.016	0.261	–0.135	–0.243	0.417	–0.060	–0.155
	*P*	0.421	0.662	0.927	0.241	0.468	0.165	0.054	0.750	0.381
Sex	*r*	–0.064	0.074	0.105	–0.107	0.089	–0.105	–0.043	–0.085	–0.132
	*P*	0.776	0.692	0.553	0.634	0.634	0.553	0.849	0.649	0.458
HR	*r*	–0.086	–0.342	–0.067	0.151	0.097	–0.167	0.059	0.117	0.238
	*P*	0.704	0.060	0.705	0.502	0.605	0.344	0.794	0.531	0.175
SBP	*r*	–0.050	0.085	–0.027	–0.301	–0.052	0.289	–0.209	–0.094	0.049
	*P*	0.824	0.648	0.878	0.174	0.781	0.098	0.352	0.615	0.781
DBP	*r*	–0.141	0.086	0.050	–0.044	–0.121	0.306	0.123	–0.126	–0.259
	*P*	0.532	0.644	0.780	0.847	0.517	0.079	0.584	0.499	0.139
Cr	*r*	0.102	0.096	0.143	–0.176	0.232	0.235	0.087	0.014	–0.245
	*P*	0.650	0.608	0.419	0.434	0.208	0.181	0.700	0.941	0.163
NT-proBNP	*r*	0.131	0.255	–0.002	–0.075	0.034	–0.056	0.070	0.035	–0.096
	*P*	0.561	0.167	0.991	0.742	0.856	0.752	0.757	0.851	0.590
Glu	*r*	–0.014	–0.214	–0.226	–0.003	–0.243	0.312	–0.154	0.333	–0.145
	*P*	0.952	0.247	0.200	0.990	0.188	0.073	0.495	0.067	0.414
TGs	*r*	0.282	–0.089	–0.173	–0.049	–0.211	0.244	–0.032	–0.298	–0.241
	*P*	0.204	0.634	0.328	0.828	0.254	0.164	0.889	0.014	0.169
TC	*r*	0.224	–0.071	–0.127	0.201	0.031	–0.153	–0.392	0.127	0.087
	*P*	0.315	0.703	0.473	0.369	0.870	0.389	0.071	0.495	0.624
LDL	*r*	0.391	–0.039	0.033	–0.131	0.042	0.070	–0.357	0.275	0.042
	*P*	0.072	0.834	0.852	0.561	0.824	0.694	0.103	0.134	0.813
HDL	*r*	–0.002	0.015	–0.006	–0.023	0.056	–0.248	–0.151	0.273	–0.080
	*P*	0.992	0.935	0.973	0.918	0.766	0.244	0.502	0.137	0.653
cTnI	*r*	–0.016	–0.341	–0.107	–0.037	–0.200	–0.246	–0.005	0.082	0.133
	*P*	0.945	0.060	0.546	0.869	0.281	0.160	0.983	0.662	0.453

*HR, heart rate; SBP, systolic blood pressure; DBP, diastolic blood pressure; Cr, creatinine; NT-proBNP, N-terminal pro-B natriuretic peptide; Glu, blood glucose; TG, triglyceride; TC, total cholesterol; LDL, low-density lipoprotein; HDL, high-density lipoprotein; cTnI, cardiac troponin I. r = Spearman’s rank correlation coefficient.*

### Serum Exosomal miR-21, miR-126, and PTEN Levels as Diagnostic Biomarkers for UA and AMI

**FIGURE 3:**
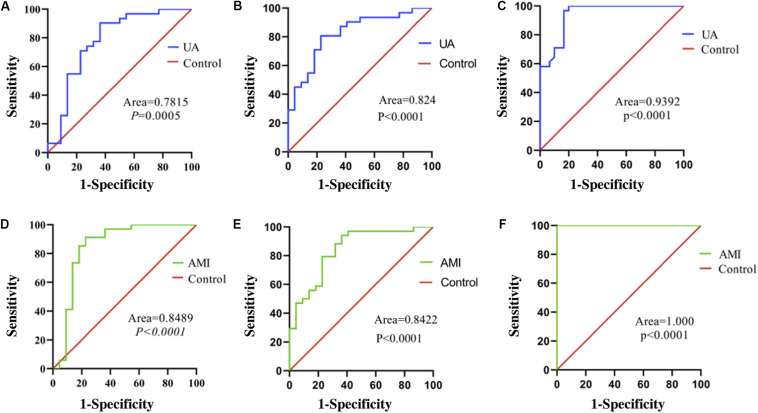
ROC curves of relative expression levels of serum exosomal miR-126, miR-21, and PTEN. **(A)** Expression of miR-126 in patients with UA. **(B)** Expression of miR-126 in patients with AMI. **(C)** Expression of miR-21 in patients with UA. **(D)** Expression of miR-21 in patients with AMI. **(E)** Expression of PTEN in patients with UA. **(F)** Expression of PTEN in patients with AMI. Exo, exosomal.

The area under the receiver operating characteristic curve (AUC) of serum exosomal miR-126 was 0.7815 [standard error (SE) = 0.06989; 95% confidence interval (CI) = 0.6445–0.9185; *P* = 0.0005] in the UA group (Figure 3A) and was 0.8489 (SE = 0.06442; 95% CI = 0.7227–0.9752; *P* < 0.0001) in the AMI group (Figure 3B). The AUC of serum exosomal miR-21 was 0.824 (SE = 0.05787; 95% CI = 0.7106–0.9375; *P* < 0.0001) in the UA group (Figure 3C) and was 0.8422 (SE = 0.05471; 95% CI = 0.7350–0.9495; *P* < 0.0001) in the AMI group (Figure 3D). The AUC of serum exosomal PTEN expression was 0.9392 (SE = 0.02883; 95% CI = 0.8827–0.9958; *P* < 0.0001) in the UA group (Figure 3E) and was 1.000 (SE = 0.000; 95% CI = 1.000–1.000; *P* < 0.0001) in the AMI group (Figure 3F).

### Positive Correlation Between Serum Exosomal miR-126 Levels and Gensini Scores in Patients With UA and AMI

**FIGURE 4:**
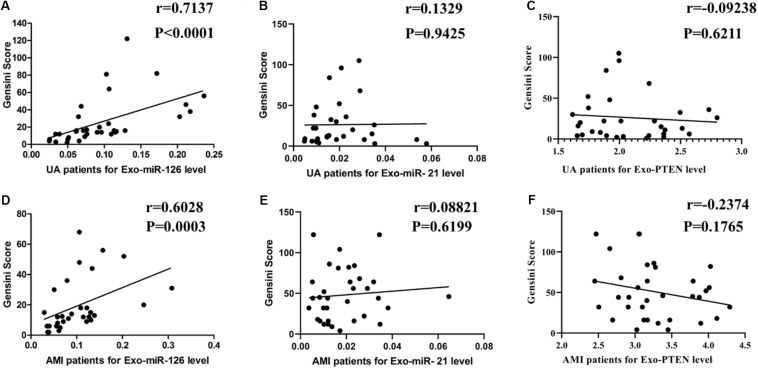
Correlation of serum exosomal miR-126, miR-21, and PTEN levels with Gensini scores. **(A)** miR-126 levels vs. Gensini scores in patients with UA. **(B)** miR-126 levels vs. Gensini scores in patients with AMI. **(C)** miR-21 levels vs. Gensini scores in patients with UA. **(D)** miR-21 levels vs. Gensini scores in patients with AMI. **(E)** PTEN levels vs. Gensini scores in patients with UA. **(F)** PTEN levels vs. Gensini scores in patients with AMI.

As shown in Figure 4, serum exosomal miR-126 levels were positively correlated with Gensini scores in patients with UA (*r* = 0.7137, *P* < 0.0001) and AMI (*r* = 0.6028, *P* = 0.0003). However, serum exosomal miR-21 levels were not related to Gensini scores in the patients with UA (*r* = 0.0133; 95% CI = −0.3427–0.3659; *P* = 0.9435) or AMI (*r* = 0.0882; 95% CI = −0.2576–0.4140; *P* = 0.6199). There was also no significant relationship between serum exosomal PTEN levels and Gensini scores in the patients with UA (*r* = −0.0924; 95% CI = −0.4326–0.2708; *P* = 0.6211) or AMI (*r* = −0.2374; 95% CI = −0.5328–0.1096; *P* = 0.1765).

## Discussion

ACS is the most serious type of coronary heart disease, and has therefore received substantial research attention in recent years to improve prevention, early diagnosis, and treatment. This effort has revealed that serum miR-499 and miR-210 can be used as biomarkers for the diagnosis of symptoms in patients with UA and non-ST segment elevation myocardial infarction within 3 h (Shalaby et al., 2016) and circulating miR-208 was suggested as a potential biomarker for the diagnosis of ACS (Wang et al., 2019). Many studies have reported exosomal miRNAs as targets for the diagnosis and treatment of various diseases, including biomarkers for hepatocellular carcinoma (Sohn et al., 2015) and Alzheimer disease (Van Giau and An, 2016).

In this study, we found that the expression of serum exosomal miR-126, miR-21, and PTEN differed between UA and AMI patients from those of healthy controls, although the direction of differences varied; miR-126 and PTEN levels were increased in the patients, whereas miR-21 levels were lower in the patients than in the controls. There was no significant difference in the expression levels of miR-21 between the UA and AMI groups, whereas PTEN and miR-126 levels were higher in the AMI patients than in the UA patients. To examine the influence of confounding factors, We used Spearman’s correlation analysis to identify potential factors associated with the expression levels of serum exosomal miR-21, miR-126, and PTEN, demonstrating no significant correlations that could affect the observed associations. Analysis of the ROC curves suggested that miR-126 and PTEN might be new candidate diagnostic biomarkers for UA and AMI, with strong ability to distinguish patients with UA and AMI from healthy people during the early stage of the disease onset. We found that the expression of serum exosomal miR-126 and PTEN had no significant correlation with NT-proBNP and blood pressure. Based on troponin may be affected by NT-proBNP and blood pressure (Kociol et al., 2010; Roffi et al., 2016) serum exosomal miR-126 and PTEN may be more specific than troponin in term of the diagnosis of AMI. In addition, our contributions lie in the discovery of biomarkers that can diagnose UA, such as serum exosomal miR-126, and PTEN. Furthermore, we found that miR-21 may be a candidate diagnostic biomarker for ACS, which could effectively distinguish ACS patients from healthy people at the early stage of disease onset.

Coronary angiography is currently the most effective method to determine the degree of coronary artery stenosis (Montalescot et al., 2013). However, this method is associated with certain drawbacks, including the invasive nature of the procedure that can be completed only in hospitals using complex machines. Furthermore, this procedure poses a great economic burden on patients and does not allow for a quick assessment of the status of coronary artery stenosis. The use of exosomal miRNA levels and recognized disease scores (such as the Gensini score) to quickly estimate the degree of coronary artery stenosis through non-invasive means would be conducive to the rapid diagnosis and treatment of patients with coronary heart disease. The Gensini score (Gensini, 1983) was established to quantify the severity and extent of coronary artery stenosis according to the blood flow in patients undergoing coronary angiography. Some studies have shown that the miR-155 level is inversely correlated with the severity of coronary artery stenosis based on the Gensini score (Zhu et al., 2014). The level of circulating miR-133a in patients with coronary heart disease was also found to be positively correlated with the Gensini score (Wang et al., 2013). We further showed that serum exosomal miR-126 levels in patients with UA and AMI were positively correlated with their Gensini scores; thus, it would be feasible to evaluate the degree of coronary artery stenosis using serum exosomal miR-126 expression as a non-invasive diagnosis modality.

However, there are still many limitations associated with this study, as factors such as blood pressure, the heart rate, creatine level, age, NT-proBNP, and other indicators must be considered. We set strict limits on these indexes when defining the exclusion criteria. Consequently, the number of subjects included in this study was substantially reduced. In future research, we will perform more comprehensive analyses of exosomes from the patient’s blood via RNA-seq and proteomics to identify more potential molecular candidates as diagnostic tools for ACS.

## Conclusion

We demonstrated that serum exosomal miR-126 and PTEN levels might serve as novel candidate diagnostic biomarkers for UA and AMI, and serum exosomal miR-21 might be a candidate diagnostic biomarker for ACS. We also found that serum exosomal miR-126 could be used to determine the severity of coronary artery stenosis in patients with UA and AMI based on the Gensini score.

## Data Availability Statement

The raw data supporting the conclusions of this article will be made available by the authors, without undue reservation, to any qualified researcher upon request.

## Ethics Statement

The studies involving human participants were reviewed and approved by the ethical committee of The Second Hospital of Jilin University. The patients/participants provided their written informed consent to participate in this study. Written informed consent was obtained from the individual(s) for the publication of any potentially identifiable images or data included in this article.

## Author Contributions

HL and CS designed the experiments. ZG collected the blood samples. YS and LZ performed the experiments and analyzed the data. HL, ZG, and CS wrote the manuscript. All authors have reviewed and approved the manuscript.

## Conflict of Interest

The authors declare that the research was conducted in the absence of any commercial or financial relationships that could be construed as a potential conflict of interest.
